# The Interplay of One-Carbon Metabolism, Mitochondrial Function, and Developmental Programming in Ruminant Livestock

**DOI:** 10.3390/jdb14010003

**Published:** 2026-01-03

**Authors:** Kazi Sarjana Safain, Kendall C. Swanson, Joel S. Caton

**Affiliations:** 1 Department of Animal Sciences, Center for Nutrition and Pregnancy, North Dakota State University, Fargo, ND 58105, USA; safai012@umn.edu (K.S.S.); kendall.swanson@ndsu.edu (K.C.S.); 2 The Hormel Institute, University of Minnesota, Austin, MN 55912, USA

**Keywords:** maternal nutrition, developmental programming, one-carbon metabolism, mitochondria, metabolomics, fetal development, ruminant livestock

## Abstract

Maternal nutrition during gestation profoundly influences fetal growth, organogenesis, and long-term offspring performance through developmental programming. Among the molecular mechanisms responsive to maternal nutrient availability, one-carbon metabolism plays a central role by integrating folate, methionine, choline, and vitamin B_12_ pathways that regulate methylation, nucleotide synthesis, and antioxidant defense. These processes link maternal nutritional status to epigenetic remodeling, cellular proliferation, and redox balance during fetal development. Mitochondria act as nutrient sensors that translate maternal metabolic cues into bioenergetic and oxidative signals, shaping tissue differentiation and metabolic flexibility. Variations in maternal diet have been associated with shifts in fetal amino acid, lipid, and energy metabolism, suggesting adaptive responses to constrained intrauterine environments. This review focuses on the molecular interplay between one-carbon metabolism, mitochondrial function, and metabolomic adaptation in developmental programming of ruminant livestock. Understanding these mechanisms offers opportunities to design precision nutritional strategies that enhance fetal growth, offspring productivity, and long-term resilience in livestock production systems.

## 1. Introduction

Early-life nutrition is increasingly recognized as a key determinant of lifelong health, physiological adaptability, and productivity across mammalian species [1,2]. During gestation, the developing fetus exists in a highly plastic state, where its growth and metabolic trajectory are shaped not only by genetic inheritance but also by the intrauterine environment. A critical driver of this environment is maternal nutrition. Even relatively minor imbalances in the supply of essential nutrients during gestation can disrupt fetal development by altering nutrient signaling, gene expression, cellular differentiation, and tissue morphogenesis. These effects are often mediated by epigenetic modifications and changes in mitochondrial function, which together influence metabolic programming, a process known as developmental programming [3]. Through this mechanism, maternal nutrition exerts a lasting influence on offspring performance, including growth rate, feed efficiency, reproductive function, and disease resistance.

In ruminant livestock, maternal nutritional challenges are common and are largely shaped by environmental variability, forage quality, grazing management, and seasonal feed availability [4]. These animals are often exposed to fluctuations in nutrient intake that coincide with critical stages of gestation. Although nutrient demands during early pregnancy may appear modest, this period is characterized by rapid cellular proliferation, embryonic patterning, placental development, and the establishment of early organ structures. As such, early gestation represents a sensitive window during which inadequate maternal nutrition can impair developmental trajectories in ways that are not immediately apparent at birth but manifest later in life as reduced productivity, reproductive inefficiencies, or metabolic disorders [5]. Addressing this issue is particularly important given the growing global demand for animal-derived foods. It is projected that by 2050, food production from the livestock sector must increase significantly to meet the dietary needs of an expanding population [6]. Ruminants, including beef and dairy cattle, sheep, and goats, are essential to this effort. They play a unique role in converting fibrous forages—grown on over 25% of the Earth’s land surface and often unsuitable for human crop production—into high-quality protein, energy, and bioavailable micronutrients [6]. In addition to producing food, ruminants contribute to agriculture, grassland ecosystem services, and rural livelihoods. Therefore, improving the efficiency, health, and sustainability of ruminant production systems is a central goal for global food security and environmental stewardship.

Developmental programming offers a promising strategy for improving ruminant livestock productivity by addressing key inefficiencies before they arise. Metabolically compromised animals—those with poor growth rates, low fertility, or suboptimal milk yield—represent significant economic losses and contribute to increased resource use per unit of production [7]. Research in humans and laboratory animals has established that developmental programming during gestation can have profound and persistent effects on offspring phenotype [8]. Although molecular evidence in ruminants has historically been limited [9,10], emerging studies in beef and Wagyu cattle demonstrate clear nutrient-responsive metabolic adaptations during fetal development [11,12,13].

Recent advances in molecular biology have opened new opportunities to investigate how maternal nutrition affects fetal development at the cellular and metabolic levels. One-carbon metabolism, mitochondrial function, and metabolomic regulation are three key biological systems that respond dynamically to nutrient availability and play central roles in developmental programming. One-carbon metabolism is vital for methylation reactions, nucleotide synthesis, and amino acid interconversion, all of which are essential for epigenetic regulation and cellular proliferation [14]. Mitochondria, as the powerhouse of the cell, regulates energy production, redox balance, and apoptosis, and are themselves sensitive to nutrient status and oxidative stress [15]. Metabolomics analyses provide a snapshot of cellular biochemical activity, allowing for the identification of metabolic shifts that reflect underlying physiological and developmental changes [16]. Understanding how these systems interact in response to maternal nutrition is critical for identifying strategies to optimize fetal development and long-term animal performance.

This literature review synthesizes current knowledge on the influence of maternal nutrition on fetal development in ruminants, with a specific focus on one-carbon metabolism, mitochondrial function, and metabolomic adaptation. By integrating current knowledge of the molecular mechanisms underlying developmental programming, this review establishes a framework for designing targeted research and nutritional interventions to improve offspring efficiency, health, and resilience in ruminant livestock.

## 2. Developmental Programming

Developmental programming refers to the long-lasting effects that maternal or early-life environmental stressors have on the growth, metabolism, and overall health of offspring. This concept, originally termed the “Barker Hypothesis” or “developmental programming”, was first proposed in human health studies linking low birth weight to increased risk of coronary heart disease in adulthood [17]. The central idea is that critical developmental windows—particularly during gestation—are highly sensitive to environmental inputs, and perturbations during these periods can induce lasting physiological and metabolic adaptations in the fetus.

In both humans and animals, adverse prenatal conditions such as poor maternal nutrition, environmental heat stress, and other physiological challenges can disrupt normal developmental trajectories. These disruptions are associated with a broad range of outcomes, including impaired fetal growth, altered tissue composition, and long-term health complications. Specifically, growth-restricted or developmentally compromised neonates show increased risk for metabolic disorders, reproductive inefficiencies, poor postnatal growth, and organ dysfunction [18]. Affected organ systems may include the brain, cardiovascular system, liver, pancreas, muscle, adipose tissue, reproductive organs, gastrointestinal tract, and immune system.

In livestock species, the implications of developmental programming are becoming increasingly recognized. Although less extensively studied compared to human models, emerging evidence suggests that compromised fetal or neonatal development in ruminants can result in increased neonatal morbidity and mortality; altered postnatal growth trajectories; unfavorable body composition (e.g., reduced muscle accretion, increased adiposity); metabolic dysfunction (e.g., impaired glucose tolerance, insulin resistance); cardiovascular anomalies; and dysfunction in key physiological systems such as the liver, intestines, and reproductive tract [19]. Numerous maternal challenges common in ruminant production such as nutrient restriction, breeding of peripubertal heifers, carrying multiple fetuses, and gestation under poor pasture conditions can compromise the fetal environment and influence long-term offspring performance. These conditions coincide with stages of rapid cell proliferation, organogenesis, and placental development, making the fetus particularly vulnerable to nutritional and environmental perturbations.

The significance of timing is critical: developmental programming occurs most prominently during “critical windows” of fetal development—periods when organs and systems are forming and differentiating. Nutritional or physiological stress during these windows can lead to lasting deficits in tissue function and metabolic regulation. Conversely, later-life interventions may have limited capacity to reverse programming-induced changes, highlighting the importance of early gestational nutrition and management [20].

In ruminants, early pregnancy is characterized by exceptionally rapid conceptus growth due to high rates of cellular proliferation [21]. During this period, before placental formation is complete, nutrient support is provided primarily through uterine histotroph—an endometrial secretion rich in amino acids, glucose, minerals and growth factors essential for embryonic survival and development [22]. As gestation progresses, the placenta becomes the key physiological interface between maternal and fetal systems, regulating nutrient exchange, gas diffusion, and waste elimination. Any compromise in placental development or function may restrict nutrient transfer and oxygen delivery, further exacerbating programming effects.

Among the many environmental inputs that influence developmental programming, maternal nutrition is among the most impactful and readily influenced environmental factor [23]. Adequate and balanced nutrient supply during pregnancy is crucial for supporting cell division, tissue differentiation, epigenetic regulation, and mitochondrial function in the developing fetus. Inadequate intake of energy, protein, or micronutrients during key windows of gestation can trigger compensatory responses that alter fetal growth patterns, endocrine signaling, and metabolic capacity—changes that often persist into adulthood. In ruminant livestock, maternal dietary restriction or imbalance has been associated with reduced fetal muscle mass, altered fat deposition, disrupted hepatic metabolism, and lower postnatal productivity [24,25].

Understanding how changes in maternal nutrition programs fetal development at molecular, cellular, and systemic levels are essential for improving animal health, reproductive efficiency, and sustainable production. As research continues to uncover the underlying mechanisms particularly involving nutrient-sensing pathways, mitochondrial bioenergetics, and epigenetic regulation, there is growing potential to apply this knowledge to precision nutritional strategies that optimize offspring development and long-term performance in livestock systems.

## 3. Maternal Nutrition as a Key Regulator of Fetal Development

Fetal survival, growth, and development are governed by a complex interplay of genetic, epigenetic, and environmental factors, among which maternal nutrition plays a central and highly influential role. Adequate maternal nutrient intake is essential for supporting embryonic and fetal development, and any disruption to maternal nutrient supply—whether due to dietary deficiencies, physiological stress, or environmental extremes—can have profound consequences on the structure and function of developing fetal tissues. Studies in animal models have demonstrated that the fetus is particularly vulnerable to nutrient imbalances during early gestation, especially during the peri-implantation period and the phase of rapid placental development [26].

In humans, deficiencies in specific nutrients such as folate, iodine, or iron have been associated with poor pregnancy outcomes, including neural tube defects, cretinism, intrauterine growth restriction (IUGR), and preterm birth [27]. Importantly, maternal undernutrition not only affects fetal outcomes but also compromises maternal health, increasing the risk of gestational complications and maternal morbidity and mortality [28]. In livestock, the influence of maternal nutrition is equally critical, particularly in ruminants, where production systems often expose pregnant animals to fluctuating nutrient availability due to variable forage quality, seasonal feed shortages, and environmental stressors. The placenta, as the key physiological interface between the dam and fetus, plays a pivotal role in mediating the effects of maternal nutrition. According to the placental nutrient-sensing model, the placenta integrates maternal and fetal signals with intrinsic nutrient-sensing pathways to regulate placental growth, nutrient transport, and maternal metabolic adaptation to gestation [29]. Additionally, the expression of imprinted genes within the placenta helps define its functional capacity during pregnancy, influencing fetal growth trajectories and postnatal outcomes. Maternal nutrient restriction—defined as any condition that reduces nutrient delivery to the fetus—can result from inadequate maternal intake, placental insufficiency, disrupted maternal metabolism, or combinations of these factors [30]. The consequences of such restriction depend greatly on the timing, severity, and duration of the insult. Early gestation, despite there being relatively low fetal nutrient demands, is a critical period for tissue differentiation, epigenetic reprogramming, and the establishment of the fetal-placental axis. Later stages of gestation, particularly the final two-thirds, are associated with rapid fetal growth and increasing nutrient demands [31].

In sheep, multiple studies have shown that maternal nutrient restriction during the final two-thirds of gestation can reduce fetal growth and result in lower birth weights [32,33]. Similar trends are observed in cattle where undernutrition in beef cattle has been associated with reduced calf birth weights and slower postnatal growth [34]. Feeding beef cows either below or above metabolizable protein requirements has also resulted in lower birth weights [35]. Interestingly, some studies report that protein supplementation during late gestation has limited effects on birth weights [36], whereas higher maternal body condition—a proxy for better nutrient status—has been correlated with increased calf birth weights [37].

While birth weight is a valuable indicator of prenatal development, it does not always reflect the full extent of programming-induced changes. Several studies have shown that maternal nutrient restriction can induce lasting alterations in postnatal metabolism, tissue composition, and endocrine function even in the absence of differences in birth weight [38]. These findings are particularly important for livestock management, as postnatal nutritional strategies are often based on herd-level averages rather than accounting for individual variation shaped by in utero conditions. Such variation may lead to management inefficiencies, unpredictable performance, and suboptimal productivity [39].

### 3.1. Consequences of Nutrient Deficiency or Imbalance

**Figure 1:**
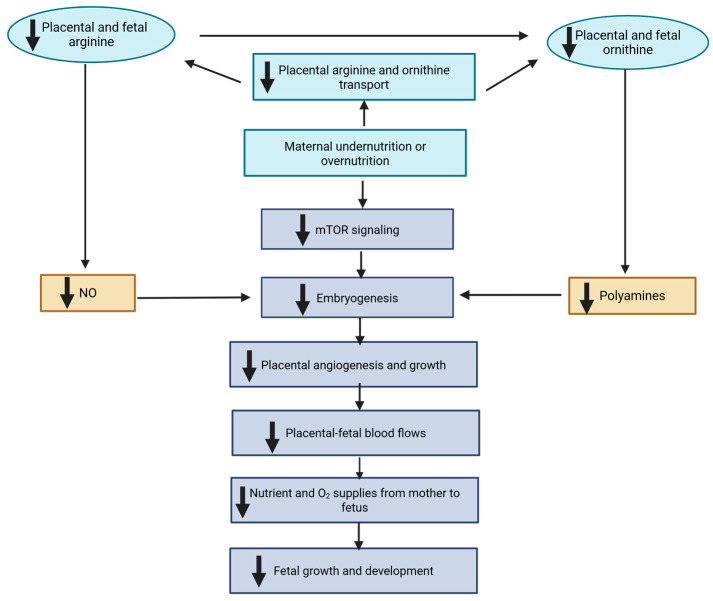
Mechanisms for fetal growth restriction in underfed and overfed dams. Both maternal undernutrition and overnutrition may impair placental syntheses of NO and polyamines, and therefore placental development and utero-placental blood flows. This may result in reduced transfer of nutrients and O_2_ from mother to fetus, and thus fetal growth restriction. mTOR, mammalian target of rapamycin. The symbol “↓” denotes reduction. Modified from Wu et al. [6].

Balanced maternal nutrition is essential for optimal fetal growth, placental development, and long-term health outcomes. Both undernutrition and overnutrition during gestation can disrupt key physiological and molecular pathways, impair fetal development, and lead to lasting postnatal consequences. These imbalances influence nutrient transfer, placental vascularization, and cellular signaling systems that regulate embryogenesis and fetal organogenesis. As illustrated in mechanistic models of maternal under- and overnutrition in Figure 1, both extremes of nutrient supply reduce placental and fetal concentrations of arginine and ornithine, amino acids critical for the synthesis of nitric oxide (NO) and polyamines. These molecules are essential for vascular development, angiogenesis, and trophoblast growth, and disruptions in their synthesis compromise placental blood flow and nutrient delivery. Mechanistically, nutrient restriction diminishes placental transport of these amino acids, downregulates nitric oxide synthase activity, and depletes co-factors such as tetrahydrobiopterin—ultimately reducing nitric oxide production. Simultaneously, reduced availability of S-adenosylmethionine (SAM) and diminished ornithine decarboxylase activity impair polyamine biosynthesis. These combined effects lead to suppression of the mTOR signaling pathway, a central regulator of nutrient sensing and cell growth, thereby further compromising placental function and embryonic development [6].

In ruminants—particularly in sheep—maternal nutrient restriction during mid to late gestation has consistently been associated with reduced fetal growth and lower birth weights [40]. These effects are even more pronounced in primiparous ewes, where nutrient competition between the dam and fetus may be heightened [41]. In cattle, although greater physiological plasticity has been observed in response to late gestational nutrient restriction—possibly due to differences in placental morphology or singleton pregnancies—calves with reduced birth weights still face increased risks. These include higher neonatal mortality, impaired ability to adapt to stressors during postnatal life, and increased vulnerability to disease and metabolic dysfunction [42]. Importantly, low birth weight alone does not always reflect the extent of fetal compromise; in many cases, postnatal growth inefficiencies and metabolic alterations occur even when birth weight appears within normal range [35]. Growth-restricted offspring often exhibit a range of long-term functional impairments, including: reduced average daily gain and gain efficiency; altered body composition (e.g., reduced lean mass, increased adiposity); delayed puberty and reduced reproductive performance; impaired glucose tolerance and insulin sensitivity; lower carcass quality and decreased lifetime productivity [36,43]. In some cases, animals that experience severe IUGR may demonstrate increased postnatal fractional growth rates, yet remain restricted in skeletal development into adulthood [44]. Such inconsistencies further underscore the limitations of using birth weight as the sole indicator of prenatal success and emphasize the need for a broader evaluation of fetal and postnatal development.

Although undernutrition has historically received the most attention, the impact of maternal overnutrition should not be overlooked. Studies using overnourished adolescent ewe models have revealed reduced offspring growth, delayed puberty, and impaired reproductive organ development [45,46]. In sheep, maternal diet-induced obesity has been associated with elevated expression of placental fatty acid transporters, leading to increased triglyceride accumulation in fetal tissues, along with heightened inflammatory signaling pathways [47]. Additional studies have reported upregulation of lipogenic genes [48], increased fat deposition in the offspring, and impairments in skeletal muscle development [49]. Similarly, in beef cattle, offspring born to non-supplemented, grazing dams displayed lower pregnancy rates, delayed reproductive maturity, and compromised carcass characteristics compared to offspring from supplemented cows [33,50]. These findings highlight the U-shaped effect of maternal nutrition, where both nutrient deficiency and excess can compromise developmental outcomes. Overall, the consequences of maternal nutrient imbalance extend well beyond birth weight and encompass systemic changes in fetal metabolism, endocrine signaling, and tissue development. These alterations reduce offspring adaptability, productivity, and longevity, ultimately impacting the efficiency and sustainability of livestock production systems. Ensuring optimal maternal nutrition across all stages of gestation is therefore critical for mitigating long-term losses and supporting productive, resilient animals in diverse production environments.

### 3.2. Timing of Nutritional Alterations Matter

**Figure 2:**
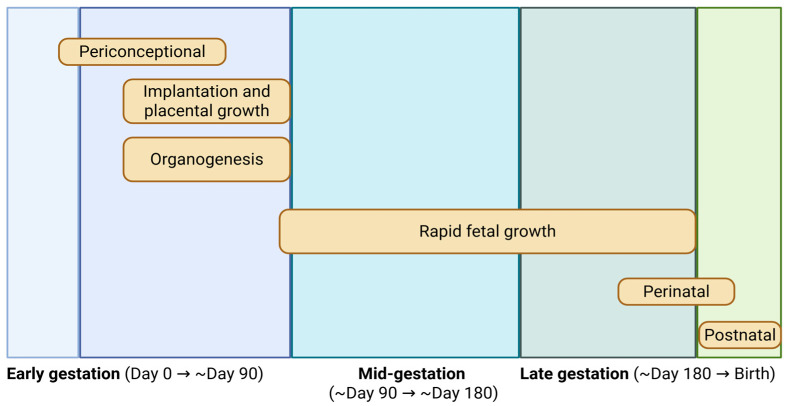
Critical windows of fetal growth and development across gestation in cattle. The schematic illustrates major developmental processes occurring from conception to birth, including the periconceptional period, early gestation (implantation, placental growth, and organogenesis), mid-gestation, and late gestation characterized by rapid fetal growth and tissue accretion. Perinatal and postnatal phases are also indicated. Approximate gestational timing is shown to highlight stage-specific sensitivity to maternal nutrition and developmental programming. Modified from Caton and Hess [30].

The stage of gestation during which maternal nutritional insults occur plays a pivotal role in determining the nature, magnitude, and persistence of developmental programming effects. Each phase of pregnancy is associated with unique developmental milestones (Figure 2), and disruptions during these windows can yield specific and often irreversible consequences for fetal growth, organogenesis, and long-term physiological function. While the total energy demands of pregnancy increase with advancing gestation, the earliest stages are particularly sensitive to maternal nutritional status. During early gestation—approximately the first 50 days in cattle—the conceptus undergoes rapid cellular proliferation and morphogenesis, transitioning from a single-celled zygote to a structurally organized embryo with developing organ systems and the formation of the placenta [27,51]. This period is marked by high tissue doubling rates and extensive cellular differentiation. Adequate nutrient supply during this phase is essential to support the foundational processes of organ development, tissue patterning, and vascularization of the uteroplacental interface [52]. Recent data indicate that maternal nutrient metabolism undergoes dynamic adaptation during gestation. For instance, a study in sheep demonstrated differential regulation of hepatic and intestinal PI3K/AKT/mTOR signaling across pregnancy stages, reflecting tissue- and stage-specific metabolic remodeling in response to gestation [53]. Nutritional deficits during early pregnancy can disrupt the formation and function of the placenta, impair critical pathways in organ development, and alter the epigenetic landscape of the fetus. Importantly, such changes may not manifest as low birth weight, yet they can result in enduring alterations in metabolic capacity, endocrine regulation, and immune competence [54]. Several studies have shown that maternal stressors—whether nutritional, metabolic, or environmental—during this early window can program offspring for reduced postnatal growth, altered glucose and lipid metabolism, and increased susceptibility to disease, independent of birth weight [55,56].

In contrast, mid-to-late gestation is characterized predominantly by rapid fetal growth and tissue accretion, particularly in metabolically active organs such as the liver, muscle, and adipose tissue [57]. This phase of gestation has received the most attention in large-animal models of developmental programming, as nutrient restriction during this time often leads to visible reductions in fetal size, altered body composition, and lower birth weights [29,37]. Compared to sheep, beef cattle appear to exhibit greater resilience to late gestational undernutrition, potentially due to species-specific differences in placental structure, nutrient partitioning, or the predominance of singleton pregnancies. The same nutritional insult can yield markedly different developmental outcomes depending on its timing within gestation. Nevertheless, both early and late gestational insults have been associated with long-term outcomes such as reduced postnatal weight gain, metabolic inefficiency, reproductive challenges, and suboptimal carcass characteristics [40]. In addition, although the various pathologies that result from developmental programming may negatively affect the health and productivity of the offspring, one of the most consequential outcomes is that these offspring may fail to express their full genetic potential. This mismatch between genotype and phenotype complicates selection accuracy and can lead to inappropriate decisions in breeding programs, ultimately hindering long-term genetic progress in livestock populations. Recent studies have strengthened these concepts. For example, nutrient restriction during mid-gestation in sheep altered fetal liver and skeletal muscle metabolites involved in amino acid, methionine, betaine, bile acid, and energy metabolism, indicating shifts in pathways linked to epigenetic regulation, protein synthesis, and glucose–lipid signaling; many of these disruptions were only partially reversed by realimentation [11]. Similarly, in beef cattle, maternal feed restriction (85% vs. 140% of metabolizable energy requirements) during mid-to-late gestation modified fetal skeletal muscle development by altering the expression of key growth, myogenic, and adipogenic genes, alongside differential DNA methylation of IGF2 and changes in miRNA abundance—despite minimal changes in fetal phenotype [12]. These findings underscore that molecular adaptations in fetal tissues can occur even in the absence of overt growth restriction, reinforcing the importance of considering both phenotypic and molecular endpoints in developmental programming research. Together, these studies provide strong evidence that mid-to-late gestation nutrient status reshapes fetal metabolic and epigenetic pathways in a tissue-specific manner. These observations underscore the critical importance of not only ensuring optimal maternal nutrition at all stages of pregnancy but also developing targeted interventions during fetal or early postnatal life to mitigate the effects of developmental insults [14,17].

### 3.3. Livestock-Specific Challenges

In livestock systems, maternal nutrition during gestation is shaped by a complex interplay of management practices, environmental pressures, physiological demands, and genetic selection. Unlike controlled experimental models, production environments introduce numerous real-world stressors that can disrupt nutrient partitioning and compromise maternal-fetal nutrient exchange. These challenges can negatively influence fetal development, reduce productivity, and impair the expression of genetic potential in offspring.

A prominent challenge in many ruminant production systems is the widespread practice of breeding young, peripubertal females, particularly in beef cattle and sheep. These animals are still amid their own postnatal growth, resulting in direct nutrient competition between maternal tissue accretion and fetal development. In such cases, the dam may prioritize nutrients for her own growth and maintenance, thereby limiting placental development and fetal nutrient availability. This scenario often leads to reduced placental efficiency, lower birth weights, and compromised offspring performance [27]. Another important factor in species like sheep and pigs is the frequent occurrence of multiple pregnancies. Uterine crowding limited placental surface area, and restricted uterine blood flow under these conditions can hinder adequate nutrient and oxygen delivery to individual fetuses. The result is often IUGR, increased perinatal mortality, and variability in postnatal performance among littermates [58]. Moreover, reduced placental mass relative to fetal demand has been linked to persistent metabolic alterations, raising the risk of later-life health and production issues.

Seasonal and environmental conditions also contribute to gestational nutritional challenges. In extensive grazing systems, breeding often occurs so that late gestation aligns with periods of low forage quality, such as winter months. During this critical window—when fetal growth accelerates—decreases in pasture quality and availability can lead to maternal undernutrition, jeopardizing fetal growth and placental function [35]. Conversely, early gestation often coincides with the hottest months of the year in many regions, and heat stress during this time can suppress maternal feed intake, reduce uterine blood flow, and impair embryonic survival [59]. Modern high-producing dairy cattle systems introduce additional complexity. These animals are genetically selected for elevated lactation performance, resulting in intense competition between milk production and fetal development during early gestation. If not properly managed, this prioritization of lactation can limit nutrient allocation to the conceptus, compromising placental growth and fetal development at a time when organogenesis and epigenetic programming are most sensitive [56]. Moreover, the increased use of assisted reproductive technologies (ART)—including embryo transfer, in vitro fertilization (IVF), and hormonal synchronization—adds another layer of risk. ART procedures can interfere with early pregnancy recognition, disrupt placental attachment, and alter uterine signaling pathways, all of which may reduce nutrient exchange and impact the programming of metabolic functions in the developing fetus [60]. Even in dams fed nutrient-sufficient diets, an inadequately primed uterine environment may contribute to long-term programming deficits in offspring.

Together, these livestock-specific challenges demonstrate that optimizing maternal nutrition during gestation is far from straightforward. Field conditions introduce multiple, often overlapping, stressors that can limit fetal development, affect offspring viability, and compromise overall production outcomes. These stressors also highlight the importance of species-specific management strategies tailored to gestational stage, environmental conditions, and production goals. Despite the significance of developmental programming in livestock, direct evidence remains limited. In contrast to human and laboratory animal models, most livestock are harvested before two years of age, limiting the ability to observe late-onset metabolic disorders that may result from prenatal insults; although a subset of animals—such as retained heifers and some bulls—may live longer, they still often go unmonitored for developmental programming outcomes. Additionally, animals with poor growth, undesirable temperament, or reproductive failure are often culled without investigation into potential in utero origins [61]. The lack of longitudinal data and comprehensive record-keeping further hinders efforts to trace developmental outcomes to gestational events.

This gap in recognition has major implications for genetic selection and herd improvement. Animals exposed to suboptimal gestational environments may not reach their full genetic potential, leading to undervaluation in breeding programs and potentially biased selection decisions. Over time, this undermines genetic progress, reduces herd productivity, and limits the overall sustainability of production systems. In the context of growing global demand for animal-sourced food, optimizing maternal nutrition and developmental programming is also vital for addressing broader issues of food security, climate resilience, and resource efficiency. Livestock contributes significantly to global protein supply and play a crucial role in rural economies. Therefore, improving developmental outcomes is not only a scientific imperative but also an economic and environmental priority. Achieving the goal of doubling livestock production by 2050 to meet population demands will require more than just genetic selection and improvements in feeding management—it will also depend on our ability to manage early life conditions that shape lifetime productivity [62].

## 4. One-Carbon Metabolism and Its Role in Fetal Development

Among various nutrient-based interventions explored to improve pregnancy outcomes, one-carbon metabolism has gained significant attention due to its central role in epigenetic regulation and cellular development. One-carbon metabolism refers to a network of interrelated biochemical pathways that integrate the folate and methionine cycles, along with transsulfuration, nucleotide biosynthesis, and polyamine production. These pathways collectively support essential physiological functions including methylation reactions, nucleotide synthesis, amino acid interconversion, redox balance, and energy metabolism [63]. Core nutrients driving one-carbon metabolites (OCM) include methionine, choline, folate, and vitamin B_12_, supported by co-factors such as vitamins B_2_, B_6_, and trace minerals like zinc, cobalt, and sulfur. The metabolite SAM, generated through methionine metabolism, serves as the universal methyl donor for DNA, RNA, and protein methylation. These methylation events are vital during early development when extensive epigenetic reprogramming occurs. In this context, OCM plays a foundational role in regulating gene expression, cellular identity, and fate determination during critical stages of embryonic and placental development [64,65]. These concepts are supported by recent comprehensive reviews demonstrating that maternal one-carbon metabolism including folate, methionine, and choline pathways play a critical regulatory role in fetal growth, placental development, and immunometabolic programming during gestation [66,67].

The demand for methyl donors is particularly high during the periconceptual and early gestational periods, when the embryo undergoes global DNA demethylation followed by targeted remethylation to establish tissue-specific gene expression patterns [68]. These epigenetic processes are highly dependent on maternal availability of OCM substrates and cofactors. Inadequate supply of OCMs during this window can disrupt normal gene regulation, leading to altered fetal growth patterns, metabolic dysregulation, and long-term consequences for offspring health and productivity.

Beyond its role in epigenetics, OCM also interacts with broader metabolic processes. For example, SAM contributes to the synthesis of polyamines, which are critical for cell proliferation, and to glutathione, the body’s primary antioxidant [64]. Additionally, OCM contributes to energy metabolism through intermediates such as succinyl-CoA in the tricarboxylic acid (TCA) cycle. Thus, disruptions in OCM can compromise not only methylation and gene regulation but also redox status, oxidative stress response, and mitochondrial function—each of which is important for proper embryonic development.

Evidence from human and rodent studies has highlighted the potential of OCM supplementation to influence developmental outcomes. In humans, the importance of folate in preventing neural tube defects (NTDs) led to widespread public health interventions. Mandatory folic acid fortification of grain products in North America, beginning in 1998, resulted in a substantial decline in NTD prevalence [69]. Today, over 80 countries have adopted similar fortification policies. However, these programs have also raised concerns about excessive intake, particularly the effects of unmetabolized folic acid, interactions with vitamin B_12_ deficiency, and potential associations with cognitive decline in the elderly [70]. These concerns underscore the importance of balance—both deficiency and excess of one-carbon nutrients can have unintended consequences.

Rodent models offer additional insights. For instance, supplementation of methyl donors (e.g., methionine, folate, B_12_, and choline) to genetically identical Agouti mice resulted in permanent alterations in DNA methylation and body composition of their offspring [68]. This outcome resulted from changes at a metastable epiallele, a genomic region where methylation is sensitive to early-life nutritional environment. These findings provided one of the first demonstrations that maternal diet can stably reprogram the epigenome, affecting offspring phenotype into adulthood [71]. Follow-up studies in humans have suggested similar effects, linking seasonal variations in one-carbon nutrient levels at conception to differential methylation at candidate epialleles [72].

Despite the growing recognition of its significance, OCM remains underexplored in ruminant livestock, particularly in the context of developmental programming. Given the importance of methylation, polyamine synthesis, and redox balance for tissue growth and metabolic regulation, strategic supplementation of OCM nutrients during gestation holds promise for enhancing reproductive efficiency and offspring health [73]. However, species-specific responses, optimal timing, and potential risks of over-supplementation remain poorly defined. As interest in precision nutrition continues to grow, understanding the mechanistic role of OCM in developmental programming across livestock species is vital. Elucidating how maternal OCM status influences placental function, epigenetic remodeling, and organ development could pave the way for nutritional interventions that improve offspring productivity, longevity, and adaptability in diverse production systems.

### 4.1. Importance for Methylation and Cell Growth

One-carbon metabolism is a critical biochemical network that supports a range of cellular processes essential for embryonic development, including DNA and histone methylation, nucleotide synthesis, amino acid interconversion, and redox homeostasis. These functions are especially important during fetal development when rapid cell division, tissue differentiation, and large-scale epigenetic reprogramming occur [74]. Central to OCM are the folate and methionine cycles, which work in tandem to generate SAM—the universal methyl group donor used in nearly all methylation reactions. Within the methionine cycle, dietary methionine is converted to SAM via the enzyme methionine adenosyltransferase (MAT). SAM donates its methyl group to a wide array of substrates—including DNA, RNA, proteins, and lipids—through the action of methyltransferases. Following methylation, S-adenosylhomocysteine (SAH) is produced and then hydrolyzed to homocysteine, which can either be recycled back into methionine or shunted into the transsulfuration pathway to produce glutathione, a key antioxidant [75].

The continuous supply of SAM depends on adequate maternal intake of methyl donors and cofactors, including methionine, choline, folate, and vitamin B_12_, as well as vitamins B_2_ and B_6_ and trace elements like zinc and sulfur. Deficiencies in any of these components can disrupt methylation capacity, leading to global hypomethylation or inappropriate locus-specific methylation, both of which are known to impair cell differentiation, organogenesis, and metabolic programming. During early gestation, the developing embryo undergoes dramatic waves of epigenetic remodeling. After initial genome-wide demethylation, de novo methylation patterns are established to guide lineage-specific gene expression. This process is highly dependent on the flux of methyl groups provided by OCM [16]. An insufficient supply of these methyl groups during this sensitive period may lead to persistent alterations in gene expression and functional deficits in key tissues such as the liver, muscle, and placenta, which could manifest as metabolic dysfunction, altered body composition, and impaired postnatal growth efficiency.

In addition to methylation, OCM contributes directly to cell proliferation through nucleotide biosynthesis, which is essential for DNA replication and repair, especially during rapid embryonic cell division. Moreover, the transsulfuration pathway that branches from OCM supplies cysteine, the rate-limiting substrate for glutathione synthesis, thereby supporting fetal antioxidant defense during periods of heightened oxidative stress. Recent research highlights the importance of maintaining a stoichiometrically balanced OCM for optimal developmental outcomes. A study investigating epigenetic modifier (EM) supplementation in bovine embryonic fibroblasts revealed that combining methionine, choline, folate, and vitamin B_12_ enhanced cell proliferation, mitochondrial respiration, and DNA methylation dynamics—even under divergent glucose conditions. These EMs supported increased mitochondrial maximal respiration and reserve capacity, particularly under moderate supplementation (2.5× and 5× the basal level), with the most significant improvements seen in cells cultured under high-glucose conditions. Interestingly, excessive supplementation (10×) of EM did not confer additional benefits and in some cases reversed the positive effects [76], emphasizing the importance of dosage and metabolic balance. Transcriptomic and methylation analyses from the same study showed that EM supplementation altered the expression and methylation of genes associated with growth regulation, energy metabolism, protein modification, and signal transduction pathways—suggesting that EMs can exert systemic effects beyond methylation alone. These findings reinforce the interconnectedness of OCM with mitochondrial bioenergetics, GTPase activity, and other cellular regulatory systems critical for embryonic development and developmental programming.

Collectively, these findings underscore that maternal one-carbon nutrient supply is vital not only for supporting fetal methylation machinery but also for facilitating mitochondrial function, cellular proliferation, and oxidative stress defense. Given the sensitivity of the developing embryo to disruptions in methylation and energy metabolism, ensuring adequate and balanced provision of OCM substrates during pregnancy may significantly enhance offspring health, growth, and long-term productivity in livestock systems.

### 4.2. One-Carbon Nutrient Supplementation Studies in Livestock Throughout Gestation

While OCM has been widely explored in human health and biomedical research, its application in livestock, particularly in the context of maternal-fetal interactions, has only recently gained attention. In ruminants, early gestation is a critical window for developmental programming, marked by rapid organogenesis, cellular differentiation, and extensive epigenetic reprogramming. During this period, the embryo experiences genome-wide DNA demethylation followed by de novo remethylation, processes that rely heavily on the availability of methyl donors and cofactors derived from one-carbon metabolism [77].

Recent studies in beef heifers have begun to shed light on how maternal nutritional status and one-carbon metabolite availability during early pregnancy influence fetal development. For example, nutrient restriction and OCM supplementation during the first 50 days of gestation altered the concentrations of vitamin B_12_ and folate in maternal jugular serum, allantoic fluid, and amniotic fluid. Interestingly, restricted heifers had elevated B_12_ and folate levels in fetal fluids, potentially as a compensatory mechanism. However, these changes were accompanied by altered hepatic gene expression in the fetus, including differential expression of key OCM enzymes such as dihydrofolate reductase and protein arginine methyltransferase [78]. Also, supplementation with a combination of OCM (methionine, choline, vitamin B_12_ and folate) up to day 63 of gestation modulated fetal small intestinal development in nutrient-restricted heifers at day 161 of gestation. Although intestinal weight was reduced in OCM-supplemented groups, vascular signaling (e.g., *VEGFR2* expression) was enhanced and cellular proliferation in the intestinal crypts was decreased [79], suggesting a physiological adaptation to nutrient stress and a potential role of OCM in fetal tissue remodeling. These findings indicate that the impact of OCM supplementation may extend beyond growth metrics to include tissue-specific functional outcomes. Earlier transcriptomic analyses from this study showed that fetal liver tissues from restricted than control dams showed that genes involved with amino acid metabolism, histone modification, and other epigenetic regulators were significantly altered by maternal diet. Importantly, nutrient restriction and supplementation appeared to partially restore the expression of genes involved in methylation and nutrient sensing pathways, as well as support the growth of key organs such as the heart and skeletal muscle [80,81]. This suggests that OCM plays a central role in regulating fetal metabolic programming and tissue development, especially during suboptimal maternal nutrition.

Additional studies have examined OCM supplementation beyond early gestation. For example, methionine supplementation during mid-pregnancy increased circulating methionine and taurine concentrations in cows, while decreasing levels of serine and glycine. Notably, a sex-specific effect was observed: male calves born to methionine-supplemented dams had higher birth weights compared to both female counterparts and offspring from control dams [82]. However, results from supplementation during late gestation have been more variable. Several studies reported no significant improvements in maternal body condition, calf birth weight, or weaning weight following methionine supplementation in the form of rumen-protected products or methionine hydroxyl analogs [83,84]. Although phenotypic responses have been inconsistent, late-gestation supplementation has elicited molecular changes in dairy calves, with ethyl-cellulose rumen-protected methionine modifying neonatal hepatic transcriptomes and OCM-related nutrient-signaling pathways [85].

Despite the lack of consistent changes in growth outcomes, some positive effects on lactational and metabolic parameters have been noted. For instance, Redifer and colleagues observed higher milk fat and solids at one week postpartum in cows receiving late-gestation methionine supplementation [86]. Similarly, calves born to methionine-supplemented dams showed enhanced average daily gain, higher body weights, elevated IGF-1 concentrations, and improved immune responses to vaccination [79]. These findings point to a potential programming effect of maternal methionine on postnatal immune competence and metabolic resilience. From a nutrient metabolism standpoint, supplementing late gestation cows with both urea and rumen-protected methionine has shown mixed outcomes. Some studies reported improved nitrogen retention and utilization efficiency [87], although broader improvements in digestibility and performance have not been consistently observed. Overall, while still emerging, the livestock literature supports the idea that strategic supplementation with OCMs during early pregnancy may mitigate the effects of maternal nutrient restriction, enhance developmental programming, and improve organ-specific development in the fetus.

**Figure 3:**
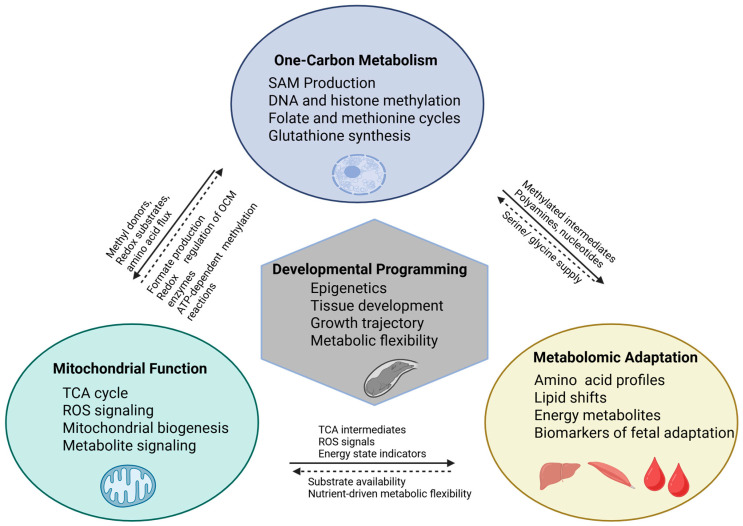
Conceptual model illustrating the integrated interplay among one-carbon metabolism, mitochondrial function, and metabolomic adaptation in shaping developmental programming in ruminants. Cartoon elements indicate the primary cellular or tissue contexts in which these processes occur. One-carbon metabolism provides methyl donors, redox substrates, and one-carbon units that support DNA and histone methylation, antioxidant defense, and substrate supply for mitochondrial metabolism. Mitochondria generate metabolic intermediates (e.g., formate and TCA cycle intermediates), ROS signals, and ATP required for methylation reactions, thereby regulating OCM enzyme activity and methylation potential. Metabolomic profiles reflect the combined activity of these pathways and provide biochemical indicators of fetal adaptation to maternal nutrient supply. Together, these integrated processes influence epigenetic regulation, tissue development, growth trajectory, and metabolic flexibility during fetal life. Solid arrows represent direct mechanistic interactions (e.g., metabolite exchange, substrate flow, or enzymatic regulation). Dashed arrows represent indirect or regulatory feedback interactions, including redox-mediated control, nutrient-sensing mechanisms, or adaptive metabolic responses.

Although one-carbon metabolism is often discussed independently, it directly interfaces with mitochondrial bioenergetics and fetal metabolite homeostasis. Many OCM substrates such as serine, glycine, and formate are generated or used within mitochondria, linking OCM flux to TCA cycle activity and ATP production [88]. Methylation capacity (SAM:SAH) also influences mtDNA methylation and mitochondrial biogenesis, while transsulfuration-derived GSH supports mitochondrial antioxidant defense [89]. These interactions demonstrate that any alteration in maternal OCM supply affects mitochondrial function and downstream fetal metabolomic signatures as illustrated in Figure 3.

## 5. Mitochondrial Function in Fetal Development and Programming

Mitochondria are maternally inherited organelles that play a central role in energy metabolism, cellular signaling, and developmental programming. As semi-autonomous structures with their own genome, mitochondria convert nutrients into ATP through oxidative phosphorylation (OXPHOS), a process tightly linked to the electron transport chain (ETC) and fueled by TCA cycle and β-oxidation pathways. Electrons derived from carbohydrate and fatty acid metabolism are transferred through ETC complexes, creating a proton gradient that drives ATP synthesis. The efficiency of this coupling determines mitochondrial bioenergetic capacity and, by extension, the energy available to support cellular differentiation, growth, and organogenesis during fetal development [90,91].

Beyond energy production, mitochondria serve as key regulators of intracellular signaling, redox balance, calcium homeostasis, and apoptosis. They generate reactive oxygen species (ROS), metabolic intermediates (e.g., α-ketoglutarate, acetyl-CoA), and high-energy compounds that can directly influence gene expression by altering chromatin structure or modifying transcription factor activity. Mitochondria thus act as cellular sensors in tissue, and transducers of nutritional or physiological signals into long-term cellular responses. These responses are mediated, in part, through epigenetic modifications such as DNA and histone methylation, with mitochondrial redox status and ROS production directly influencing these processes [92].

Given this central role of the mitochondria in regulating cellular function, disruptions in mitochondrial function during gestation can have profound implications for developmental programming. One compelling example in IUGR fetal sheep showed that hepatic mitochondrial dysfunction was associated with metabolic maladaptation [93]. Transcriptomic and metabolomic profiling of IUGR fetal livers under insulin-clamp conditions revealed upregulation of gluconeogenic genes (e.g., TRB3, SGK1, MYC) and suppression of genes involved in lipid catabolism, amino acid degradation, and mitochondrial energy production. These alterations contributed to increased hepatic glucose production, insulin resistance, and reduced energy state, hallmarks of mitochondrial inefficiency and stress [94]. The study also identified elevated intracellular amino acid concentrations and increased cellular stress, suggesting mitochondrial impairment contributes to altered fetal nutrient sensing and energy homeostasis. In Wagyu cattle fetuses, maternal nutrient restriction is associated with altered expression of oxidative phosphorylation–related genes and coordinated shifts in hepatic metabolite–transcript networks involved in central energy metabolism, supporting mitochondria-linked metabolic adaptation during developmental programming [95]. Another example involves multigenerational programming of mitochondrial dysfunction following maternal overnutrition. In a mouse model, females fed a high-fat/high-sucrose diet before conception transmitted mitochondrial abnormalities not only to their F1 offspring, but also through the F2 and F3 generations. These offspring exhibited impaired skeletal muscle insulin signaling, altered expression of ETC complex proteins, and disrupted mitochondrial dynamics. Intriguingly, mitochondrial defects were detected in the oocytes of F1 and F2 generations, indicating a potential mechanism of transgenerational transmission via germline mitochondria [96]. This study illustrates how maternal dietary environments can have lasting effects on mitochondrial function, independent of the postnatal diet, and reinforces the idea that early-life developmental programming has long-term metabolic implications. Together, these findings underscore mitochondria as central nodes in the developmental origins of health and disease.

Additionally, mitochondria readily respond to OCM-derived substrates, including SAM, folate-linked one-carbon units, and redox cofactors, which directly influence mitochondrial respiration, antioxidant capacity, and metabolic signaling [97]. In turn, mitochondria regulate OCM activity through redox balance, formate production, and ROS-mediated control of methylation reactions. Mitochondrial dysfunction alters levels of TCA intermediates, acylcarnitines, and redox metabolites [98], which may also affect nutrient-restricted ruminant fetuses. These metabolite shifts feed back into OCM by modifying serine, glycine, and methionine availability as well as the cellular methylation potential [99]. Thus, mitochondrial bioenergetics act as a central integrator linking maternal nutrient supply, OCM flux, and the metabolomic phenotype underlying developmental programming. Because these mitochondrial and epigenetic alterations are established during critical developmental windows [100], the resulting metabolic signatures may persist into postnatal life and, and also, may extend into subsequent generations through maternal inheritance.

## 6. Metabolomics as a Tool to Study Fetal Development

Metabolomics is one of the most recent and promising advances in the “omics” sciences, offering a powerful means to characterize the complete set of small molecules—typically less than 1 kDa—in cells, tissues, or biological fluids [101]. Because metabolites are the downstream products of gene expression and enzymatic activity, metabolomics provides a representation of the actual biochemical status of a biological system. This makes it particularly valuable for studying fetal development, where subtle alterations in nutrient availability or maternal physiology can trigger profound changes in fetal metabolic pathways. Unlike genomics or transcriptomics, which reflect genetic potential or regulatory activity, metabolomics provides a snapshot of the physiological state of cells or tissues at a given point in time. This functional output enables researchers to detect early metabolic shifts related to nutrient restriction, oxidative stress, or mitochondrial dysfunction. As such, metabolomics is well-suited for identifying metabolic alterations associated with developmental programming, particularly those influenced by maternal diet during gestation, and can serve as a valuable tool to generate hypotheses for more targeted investigations into underlying molecular mechanisms. In livestock research, metabolomics has provided insight into identifying fetal adaptations to maternal nutrition. For example, metabolomic analysis of fetal liver at day 83 of gestation revealed increased concentrations of metabolites related to carbohydrate, lipid, and energy metabolism in fetuses from dams fed low vitamin diets and experiencing reduced gain [102,103]. These findings suggest either a sparing of fetal hepatic metabolism or an adaptive reduction in metabolic activity in response to decreased nutrient and OCM availability. In fetal liver and skeletal muscle, comprehensive LC–MS/MS analyses have shown that maternal undernutrition alters amino acid, carbohydrate, lipid, and transsulfuration/methionine pathways in sheep, with several of these changes partially reversible following maternal realimentation. Notably, metabolites involved in methionine, betaine, bile acid, and urea cycle metabolism were consistently altered, suggesting potential downstream consequences for epigenetic regulation, protein synthesis, and mitochondrial energy metabolism. These findings indicate that fetal metabolic plasticity is highly sensitive to maternal nutrient supply during early-to-mid gestation [104]. Other studies have demonstrated that maternal protein-energy supplementation during gestation alters the plasma metabolome of both cows and their calves. In cows, differences in metabolites such as taurine, glutamic acid, and histidine were observed between early gestation and late pregnancy. In calves, distinct shifts in metabolites like carnosine and alanine were identified, suggesting that prenatal nutritional strategies can shape postnatal metabolic profiles. Enrichment analyses pointed to changes in amino acid metabolism including histidine and beta-alanine metabolism as key pathways affected by developmental programming, potentially indicating epigenetic influences are carried into early postnatal life [105,106].

Together, one-carbon metabolism, mitochondrial function, and metabolomic adaptation form an integrated regulatory network that mediates the effects of maternal nutrition on fetal development, as illustrated in Figure 3. OCM supplies methyl donors, folate-derived one-carbon units, and redox substrates that sustain mitochondrial energy metabolism, antioxidant capacity, and methylation reactions [107]. Mitochondria, in turn, generates formate, ROS, and TCA cycle intermediates that modulate OCM enzyme activity and influence cellular methylation [108,109]. The resulting metabolomic profile represents the combined activity of these pathways and serves as a biochemical signature of fetal adaptation to nutrient availability [88,110]. This interconnected framework underscores that developmental programming in livestock arises not from independent pathways but from coordinated interactions among epigenetic regulation, mitochondrial bioenergetics, and metabolic signaling.

## 7. Integrated Mito-Metabolomics Perspective in Maternal-Fetal Research

“Omics,” defined as the large-scale analysis of data representing the structure and function of biological systems at a particular molecular level, has substantially transformed how we interrogate complex biological processes. These top-down approaches, which emerged with the advancement of omics technologies, have enabled researchers to move beyond single-pathway approaches. When coupled with bottom-up strategies, omics offers a holistic framework for efficiently exploring biological systems [111]. However, traditional single-omics approaches—such as metabolomics or transcriptomics in isolation—provide only a fragmented view of the regulatory mechanisms governing fetal development [112]. While informative, these siloed datasets often fail to capture the dynamic interplay between gene expression, enzymatic activity, mitochondrial energetics, and metabolic output. In contrast, multi-omics integration, particularly approaches that combine mitochondrial function assessments with metabolomics (“mito-metabolomics”), might enable a more comprehensive understanding of how nutrient availability and cellular energy systems intersect to influence fetal developmental programming.

Recent studies underscore the significance of integrating metabolomic and transcriptomic analyses to elucidate the effects of maternal health on fetal development. For instance, research by Stuart et al. demonstrated that maternal obesity in mice leads to alterations in the maternal plasma metabolome and changes in gene expression pathways critical for placental development, highlighting the profound impact of maternal metabolic status on early embryonic growth [113]. Tissue-specific metabolomic and epigenetic adaptations to maternal nutrient restriction have been reported across fetal organs in beef cattle, reinforcing mitochondria as a central regulator of fetal metabolic programming. In Wagyu cattle, nutrient restriction during mid-to-late gestation altered growth-, myogenic-, and adipogenic-gene expression in a muscle-dependent manner, with the longissimus dorsi exhibiting greater transcriptional, DNA-methylation, and microRNA sensitivity than the semitendinosus muscle, despite minimal differences in gross fetal phenotype [114]. Beyond skeletal muscle, integrated metabolomic, transcriptomic, and epigenomic analyses demonstrate coordinated metabolic remodeling in fetal liver and immune tissues. In Wagyu and Japanese Black cattle, maternal undernutrition altered fetal liver metabolites associated with amino acid, TCA cycle, glucuronidation, lipid, and urea-cycle pathways, accompanied by suppression of genes involved in glucose homeostasis, lipid metabolism, and steroid regulation. Parallel epigenomic analyses revealed that changes in histone H3K4me3—rather than promoter DNA methylation—were closely linked to differential expression of stress-response, lipid-handling, and energy-regulatory genes, highlighting histone methylation as a key mediator of nutrient-responsive hepatic adaptation [115]. Collectively, these multi-omics studies demonstrate that maternal nutrient restriction drives coordinated mitochondrial, metabolic, and epigenetic adaptations across fetal tissues, supporting the concept that developmental programming arises from integrated bioenergetic and regulatory networks rather than isolated molecular pathways. Similarly, studies have shown that maternal obesity is associated with metabolic perturbations early in pregnancy, which may influence fetal growth trajectories [116]. These findings further emphasize the value of a multi-omics approach in understanding the complex interplay between maternal health and fetal development. These instances underscore a central limitation of single-omics studies: while they can detect associations, they often cannot uncover causal or coordinated mechanisms. For instance, metabolomics may reveal elevated glutathione levels, but without mitochondrial data, it is unclear whether the change is driven by increased oxidative stress, disrupted substrate flux, or impaired ATP synthesis. Conversely, mitochondrial respiration assays might show reduced coupling efficiency, yet without metabolite context, the origin and biological implications of the dysfunction remain uncertain. In parallel, recent human-based fetal and neonatal studies have begun to explore how multi-layered regulation including epigenetics and transcriptomics shapes development, particularly in the immune system. In neonates exposed to chorioamnionitis, an intrauterine inflammatory condition, chromatin immunoprecipitation sequencing revealed widespread remodeling of histone modifications, particularly increased H3K4me3 enrichment in intronic and intergenic regions. These changes were associated with suppressed immune gene expression and a diminished inflammatory response to secondary challenges contributing to a heightened risk of neonatal sepsis [117]. Additional studies have shown that as gestation progresses toward term, activating histone marks (H3K4me3) accumulate at promoters of key pro-inflammatory cytokines (e.g., IL1B, IL6, TNF), whereas preterm neonates exhibit lower promoter accessibility and decreased expression of these genes [118]. These findings illustrate how epigenetic regulation interacts with transcriptional activity during critical windows of fetal development and highlight the importance of integrating omics data with functional and epigenomic data to understand how environmental stressors, such as infection or malnutrition, influence long-term outcomes.

Despite these advancements, studies combining metabolomics with mitochondrial bioenergetics and epigenomic data during gestation in ruminants, specifically in cattle remain very limited. Most studies continue to rely on single-omics approaches [119,120]—such as transcriptomics or metabolomics—without incorporating complementary functional measures such as mitochondrial respiration, epigenetic profiling, or chromatin state analysis. This narrow focus limits our understanding of the coordinated biological networks that mediate developmental programming. Given the complex physiological interactions among nutrient sensing, mitochondrial function, and organ development, especially under conditions of maternal nutrient restriction, there is a critical need for using integrated approaches in ruminant research.

## 8. Conclusions

Maternal nutrition exerts profound influence on fetal development through interconnected mechanisms involving one-carbon metabolism, mitochondrial function, and metabolic adaptation. While the significance of these regulatory mechanisms has been established in non-ruminant species, comparable molecular evidence in ruminants remains limited. This gap is especially evident during early and mid-gestation, when organogenesis, placental development, and epigenetic remodeling are most sensitive to nutrient supply. Current livestock research has largely emphasized phenotypic outcomes such as birth weight or postnatal growth, offering minimal insight into the underlying metabolic and epigenetic processes that shape these traits. A more integrated understanding of how maternal micronutrients, mitochondrial bioenergetics, and fetal metabolic pathways interact is essential to clarify how developmental programming occurs in cattle and other ruminants. Advancing this mechanistic knowledge through multi-omics and functional approaches will enable the development of targeted nutritional strategies to improve offspring health, production efficiency, and long-term resilience in livestock systems.

## Data Availability

No new data were generated in this review. All referenced data are from previously published studies (see citations), and permissions for reuse have been secured from the original copyright holders.
